# Parkinson’s disease detection from 20-step walking tests using inertial sensors of a smartphone: Machine learning approach based on an observational case-control study

**DOI:** 10.1371/journal.pone.0236258

**Published:** 2020-07-23

**Authors:** Milla Juutinen, Cassia Wang, Justin Zhu, Juan Haladjian, Jari Ruokolainen, Juha Puustinen, Antti Vehkaoja

**Affiliations:** 1 Faculty of Medicine and Health Technology, Tampere University, Tampere, Finland; 2 Department of Electrical Engineering and Computer Science, Massachusetts Institute of Technology, Cambridge, Massachusetts, United States of America; 3 School of Engineering and Applied Sciences, Harvard University, Cambridge, Massachusetts, United States of America; 4 Department of Informatics, Technical University of Munich, Munich, Germany; 5 Faculty of Management and Business, Tampere University, Tampere, Finland; 6 Tampere University of Applied Sciences, Tampere, Finland; 7 Unit of Neurology, Satakunta Hospital District, Pori, Finland; 8 Clinical Pharmacy Group, University of Helsinki, Helsinki, Finland; 9 Department of Neurology, University of Turku, Turku, Finland; Cardiff University, UNITED KINGDOM

## Abstract

Parkinson’s disease (PD) is a neurodegenerative disease inducing dystrophy of the motor system. Automatic movement analysis systems have potential in improving patient care by enabling personalized and more accurate adjust of treatment. These systems utilize machine learning to classify the movement properties based on the features derived from the signals. Smartphones can provide an inexpensive measurement platform with their built-in sensors for movement assessment. This study compared three feature selection and nine classification methods for identifying PD patients from control subjects based on accelerometer and gyroscope signals measured with a smartphone during a 20-step walking test. Minimum Redundancy Maximum Relevance (mRMR) and sequential feature selection with both forward (SFS) and backward (SBS) propagation directions were used in this study. The number of selected features was narrowed down from 201 to 4–15 features by applying SFS and mRMR methods. From the methods compared in this study, the highest accuracy for individual steps was achieved with SFS (7 features) and Naive Bayes classifier (accuracy 75.3%), and the second highest accuracy with SFS (4 features) and k Nearest neighbours (accuracy 75.1%). Leave-one-subject-out cross-validation was used in the analysis. For the overall classification of each subject, which was based on the majority vote of the classified steps, k Nearest Neighbors provided the most accurate result with an accuracy of 84.5% and an error rate of 15.5%. This study shows the differences in feature selection methods and classifiers and provides generalizations for optimizing methodologies for smartphone-based monitoring of PD patients. The results are promising for further developing the analysis system for longer measurements carried out in free-living conditions.

## Introduction

Parkinson’s disease (PD) [1] is a progressive neurological disease affecting the motor ability of the patient, by inducing involuntary tremor at rest, rigidity and slowness of movement [2]. Additionally, PD may cause difficulties in walking by impacting balance and increasing the frequency of falls or cause the inability to fluently walk through visual obstacles, also known as freezing of gait [3]. The symptoms may be suppressed by levodopa medication, which compensates the loss of dopamine in the central nervous system, but the effect of the medication varies on an individual level and is dependent on many factors. PD cannot be cured, but with a successful symptom management plan, the quality of life of the patient can be maintained for decades.

The Movement Disorder Society has developed the clinical diagnostic criteria for PD, which was last revised and published in 2015 [2]. The diagnosis consists of excluding any other diseases with similar symptoms and the detection of at least two out of three cardinal features of PD. Currently the assessment of PD patients is based on subjective assessment by a neurologist, including physical tests and interviews of the patient, but not necessarily using any standard format of questionnaire or test sequence like the Unified Parkinson’s Disease Rating Scale (UPDRS) [4]. However, UPDRS is widely used in categorizing the symptoms during research studies, for example by Eskofier et al. [5] and Arora et al. [6].

As PD is a progressive disease, the patients regularly visit doctors to follow-up their health and to adjust the treatments if necessary. These follow-ups are based on subjective visual assessment by the neurologist and patient’s own description of the symptoms. However, visual assessment is prone to errors. The severity of symptoms of a PD patient changes on a daily basis due to medication intake, stress, or the overall health of the patient. Therefore, there is a need for methods to objectively assess PD symptoms for longer periods to maintain appropriate medication balance and the quality of life at home. Furthermore, up to one-fourth of diagnoses are incorrect when the analysis is only made based on the initial visit [7, 8]. Diagnostic accuracy improves during follow-ups, which also indicates that a longer period of monitoring time would help in reaching the correct diagnosis immediately. As wearable sensor development has been increasing in recent years, the option for objective assessment of patients at home without increasing the number of clinical visits has become more feasible.

Objective measurement methods have been studied and applied in detecting and monitoring PD. Wearable sensors have been used in PD to study various different motor symptoms, for example, tremor [9], rigidity, freezing of gait [10–12] and the risk of fall [13, 14] or fall detection [15, 16] both in the clinic and at home. For example, Del Din et al. [17] discovered that wearable sensors provide accurate information for the analysis of gait characteristics in free-living environments. Schlachetzki et al. [18] studied the differences of gait properties in the clinic by conducting 10 meter walking tests for 190 PD patients and 101 age-matched controls by attaching inertial sensor units to both shoes. The difference between gait parameters in PD patients and controls was significant at moderate stages of the disease. These studies show that the changes in movement can not only be observed visually but also measured quantitatively with wearable sensors.

Machine learning approaches have been popular in many areas, and the use has also increased in the research in PD application area. Several machine learning studies featuring different symptoms have been conducted with a varying number of participants [5, 10, 12, 19–23]. Most of the studies implementing machine learning in assessing PD symptoms at the laboratory or clinic environment have collected a relatively small dataset (n = 5–20) of PD patients [5, 10, 19–21]. Some have also collected a group of healthy controls [12, 22]. There is a larger study by Klucken et al. [23], in which 92 subjects were used in the training phase and 81 subjects were used in an independent validation phase. Clinic and laboratory studies have reached classification results up to 96% in detecting freezing of gait [10, 12, 21]. There are several studies reaching to accuracies of 82–90% in detecting other symptoms [5, 19, 20, 23].

In addition to the clinic measurements, few home or free-living studies have been conducted as well. A small-scale test by Arora et al. [6] showed that Random Forest (RF) is an efficient classification method in detecting PD from controls using gait. 98% sensitivity and specificity were achieved in their study. Larger datasets of several hundreds or thousands of participants have been collected by recruiting the subjects personally [24] or completely remotely using the subjects own cell phones to participate and collect the data [25, 26]. This implies that large datasets can be collected via smartphone with relatively small resources. The selection of the dataset depends on the research question, for example Nguyen et al. [27] used the data of 6805 subjects with less balanced groups, whereas Mehrang et al. [28] used only 1237 subjects of the same dataset but with age-matched PD patients and controls. They discriminated PD patients from healthy controls with RF receiving accuracy, sensitivity and specificity of 70% each. Further, the effects of taking levodopa medication have been detected with an area under the curve value of 0.7 using K-nearest neighbours [27] and accuracy of 71% using RF [26] classification methods. Body fixed sensors can also monitor for example, walk-to-sit and sit-to-walk transitions to discriminate healthy older adults from mild and severe PD patients (85–92% accuracy using Support Vector Machine -type classifier) [24].

The gaps remaining in the existing literature seem to be the following: a variety of symptoms has been assessed in clinic-based measurements, whereas most of the home measurements have currently only discriminated PD patients from healthy controls although some studies included the study of medication effects [27]. Also, some of the earlier studies are rather small in the number of participants, therefore statistically powerful datasets are needed to assess the symptoms at home using machine learning. Finally, machine learning does not have a single methodology that suits for every purpose. Therefore, testing several methods for the datasets before analyzing the details is important to find optimal methodologies for the type of data used in the study, and to gather more knowledge on which machine learning methods are the most useful in this application area.

The research question of this study was to determine, what are feasible ways of selecting the features and classifying the walking tests performed at the clinic [29]. We aimed to analyze different feature selection methods to find the most feasible features for detecting the differences in walking. We also aimed to test the performance of different machine learning algorithms to find suitable methods to differentiate the PD group and control group from each other. These research questions aim to provide more knowledge on the use of smartphones in measuring PD patients, and to provide general information of the machine learning methodologies suitable for this application area.

This article is structured as follows: Material and Methods section provides the details of the dataset used in this study, and the methods used in the human activity recognition chain. Results section provides the numerical and graphical results of the feature selection and classification. Discussion section analyzes the results compared to earlier state-of-art and discusses possible limitations of the study and future work. Finally, Conclusions section provides a summary of the study and our main conclusions.

## Material and methods

### Material

Data collection was performed in Satakunta Hospital District, Pori, Finland during 2018 [29]. 103 subjects participated in the study, and 97 subjects went through the whole study protocol. Patients and control subjects were recruited from outpatients of Satakunta Hospital District and volunteers living in the same area. Before conducting the measurements, the subjects were classified according to the Finnish Unified Parkinson’s Disease Rating Scale, section V (FIN-UPDRS-V) [4] to categories with score 0–5, 0 indicating no symptoms of PD and scores 1–5 indicating the increasing severity of PD symptoms. The subjects signed an informed consent. The study protocol was approved by the Ethics Committee of the Hospital District of Southwest Finland in Turku, Finland (ETMK 101/18012017) and the National Supervisory Authority for Welfare and Health (Valvira) in Helsinki, Finland approved the study protocol (identification number 394–2018). More details of the study protocol and ethical aspects are discussed by Jauhiainen et al. in [29].

**Fig 1:**
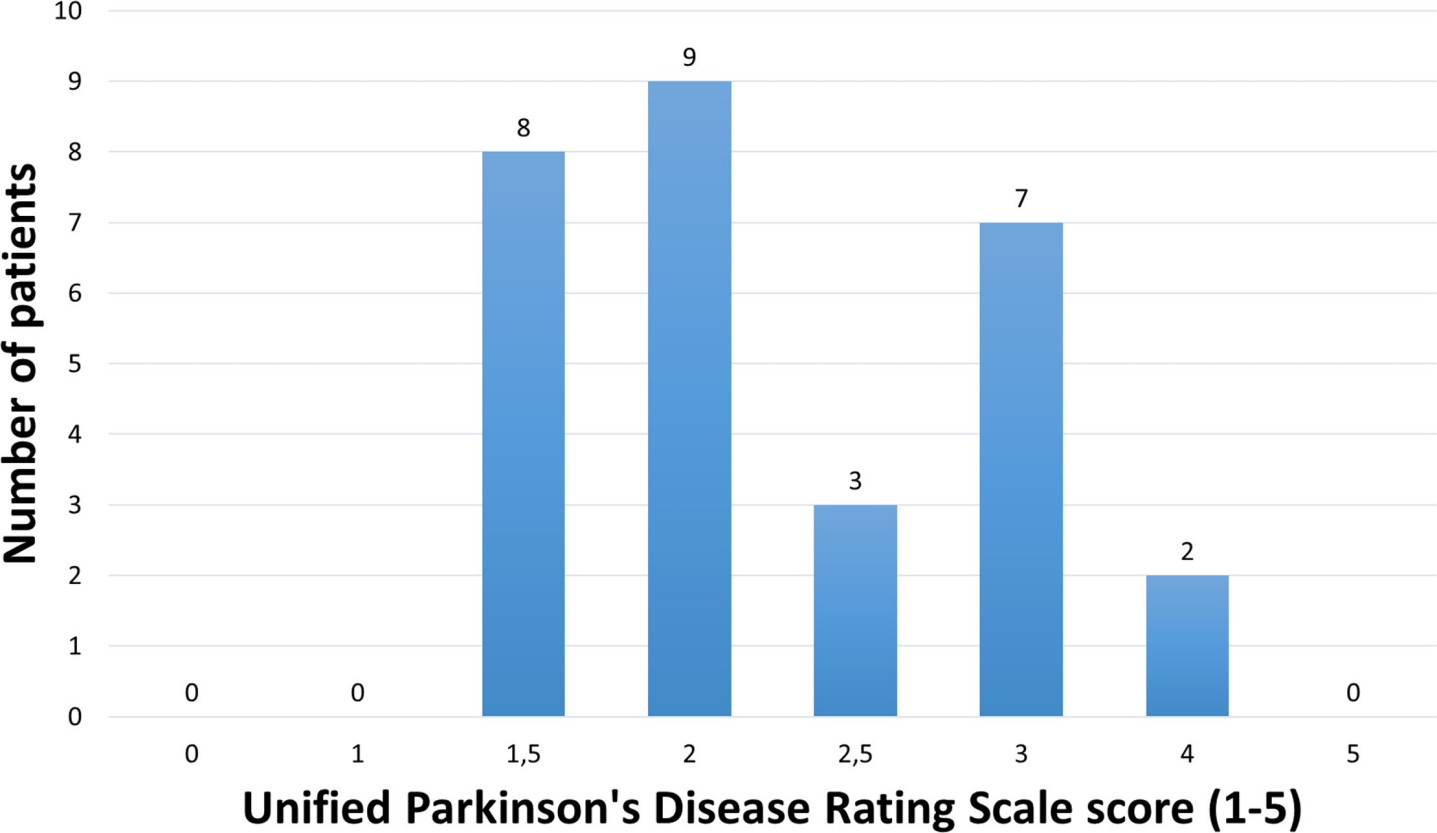
The distribution of PD patients based on the total Unified Parkinson’s Disease Rating Scale (UPDRS). The modified score is used to evaluate the symptoms, 0 being no symptoms, and 5 denoting full bed rest or using a wheelchair.

In order to optimize the performance for detecting both PD and non-PD subjects, the dataset needs to have enough training data for both groups. Thus, a balanced subset was selected from the study population: there were 29 control subjects successfully recorded at the clinic; thus we also selected 29 measured PD patients for this study. The selection criteria was the following: the PD patients were included by the severity of the symptoms in the UPDRS score. The patients with less severe symptoms were excluded from the dataset. All patients with a score of 1 (18 in total), and 4 patients with a score of 1.5 were excluded. The exclusion of these subjects during the matching phase can be justified, since the medication schedule of the PD patients is not interfered and therefore, some of the patients who took the medication just before the test had only very mild symptoms or even were asymptomatic, if they were responding well to their normal dose. Since not all patients with score 1.5 were excluded, the selection of inclusion and exclusion of those patients was random. The UPDRS score distribution of all patients in the selected dataset is seen in Fig 1.

The distribution of PD patients between scores 1.5–4 was relatively homogenous, although each subgroup is rather small to be analyzed separately. In addition, the basic background information and medical history was recorded from all the subjects. The descriptive information of the cohort is shown in Table 1.

**Table 1:** Demographic data of cohort used in study.

	Parkinson’s Disease	Control	Total
**Number of subjects**	29	29	58
**Age, Mean ± standard deviation**	69.1 ± 7.9 years	60.4 ± 14.1 years	64.8 ± 12.2 years
**Body mass index (kg/m^2), mean ± standard deviation**	26.4 ± 3.4	26.6 ± 4.0	26.5 ± 3.7
**Gender (Female)**	15	24	39
**Gender (Male)**	14	5	19
**Number of steps**	1075	1043	2118

It can be seen in Table 1 that the gender distribution of the subjects is skewed: whereas the number of PD patients is almost equal between men and women, there are many more female controls compared to the male controls. However, the other parameters are similar among the two groups, such as the mean age and the body mass index between groups. In addition, any earlier diagnoses were recorded for later use in evaluating the machine learning model performance. For example, if misclassifications occurred, the subject information could be checked for any explanatory factors affecting the walking test.

## Methods

**Fig 2:**
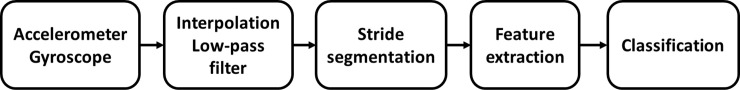
General workflow of the machine learning system from [31]. Figure adapted from the original source.

All data analysis of this study was performed with MATLAB R2018a version (The MathWorks, Inc, Natick, MA). Data analysis of this study was following the general workflow of classification studies, presented for example, by Bulling et al. [30] and later by Haladjian et al. [31]. The workflow is illustrated in Fig 2.

Walking data were collected using a smartphone inertial sensors, and the data were first preprocessed and segmented into individual strides. A large set of features, 201, were calculated from each stride, and the most appropriate features were selected to be used in the feature selection phase. Finally, the strides were classified into two classes: PD or non-PD, and the final classification of subjects was the class with majority of the strides. Each processing step is discussed in detail in the following sections.

### Data collection

A walking test was used to measure the physical condition of the subjects in addition to the subjective UPDRS assessment made by the study physiotherapist [29]. The protocol from the mPower study [25] was adopted, and each subject walked indoors in a straight hallway for 20 steps. The physiotherapist counted the steps silently and asked the subject to start and stop walking accordingly [29]. The subjects did not count their steps, since rhythmic audio or conscious counting is known to improve the walking of PD patients [32–34]. In practice, some of the subjects took an extra step after being asked to stop walking. The walking test was conducted twice for each subject, but some of the recordings were unsuccessful. Thus, each included subject had 19–41 steps in total in the analysis [29].

**Fig 3:**
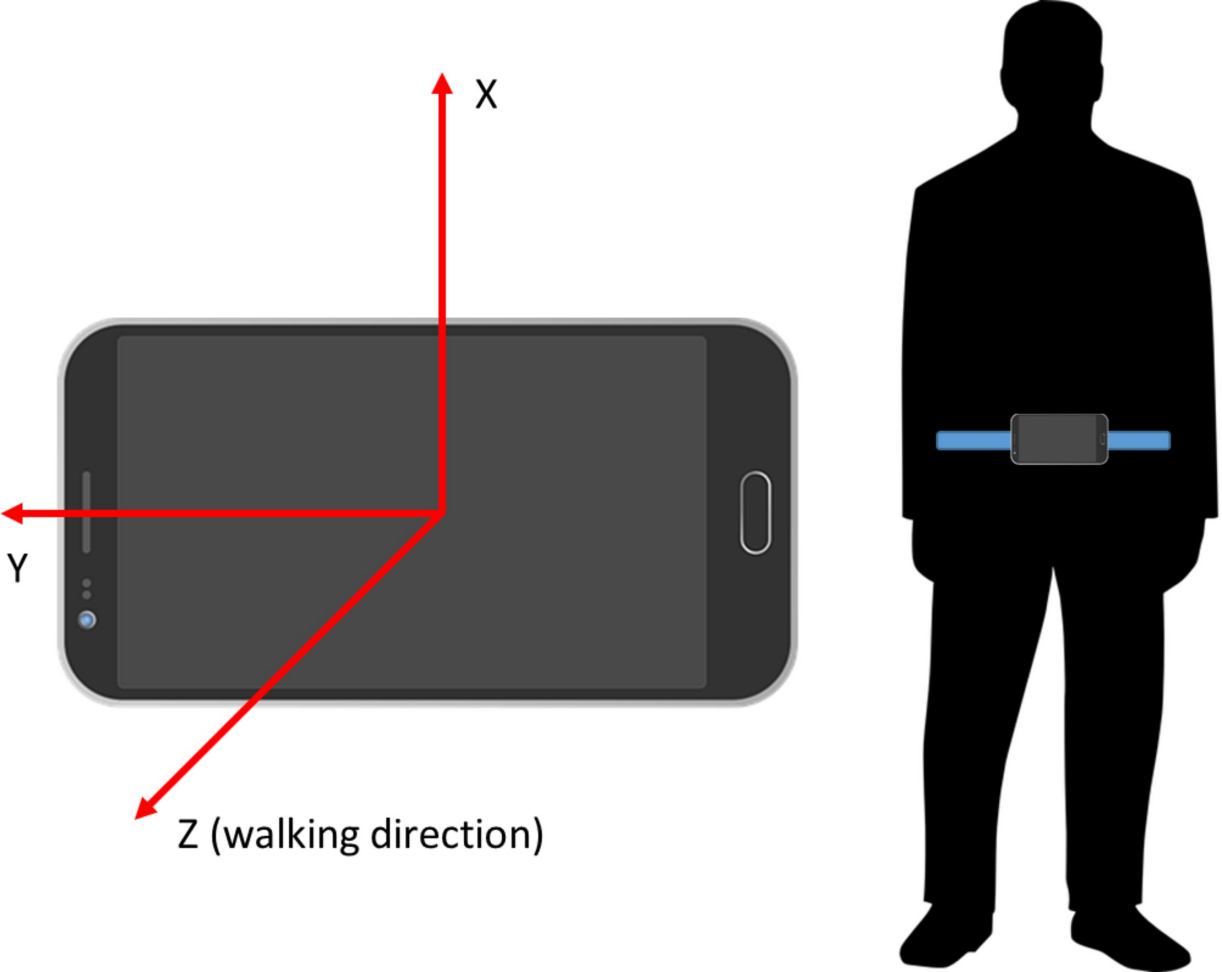
The orientation of the smartphone during 20 step walking tests: X, Y and Z axes denote up-down, right-left and forth-back directions, respectively. Positive Z axis denotes the walking direction, when walking a straight line.

During the walking tests, acceleration and gyroscope signals were collected with built-in sensors of an Android smartphone (Nokia(R) 6, with Android 8) attached firmly to subject’s waist in a bag [29]. The device orientation with the walking direction is illustrated in Fig 3.

From the subject’s perspective, The x-axis was defined to be the plane in the vertical direction (up is positive), the Y axis in the transverse direction (right is positive), and the Z axis in the anterior-posterior direction (forward is positive). Data collection was initiated and ended with another smartphone, which was used by the study physiotherapist as a controller for the data collection smartphone. The data was stored as separate files in the controller smartphone’s internal memory, and transferred manually to the database.

### Preprocessing

**Fig 4:**
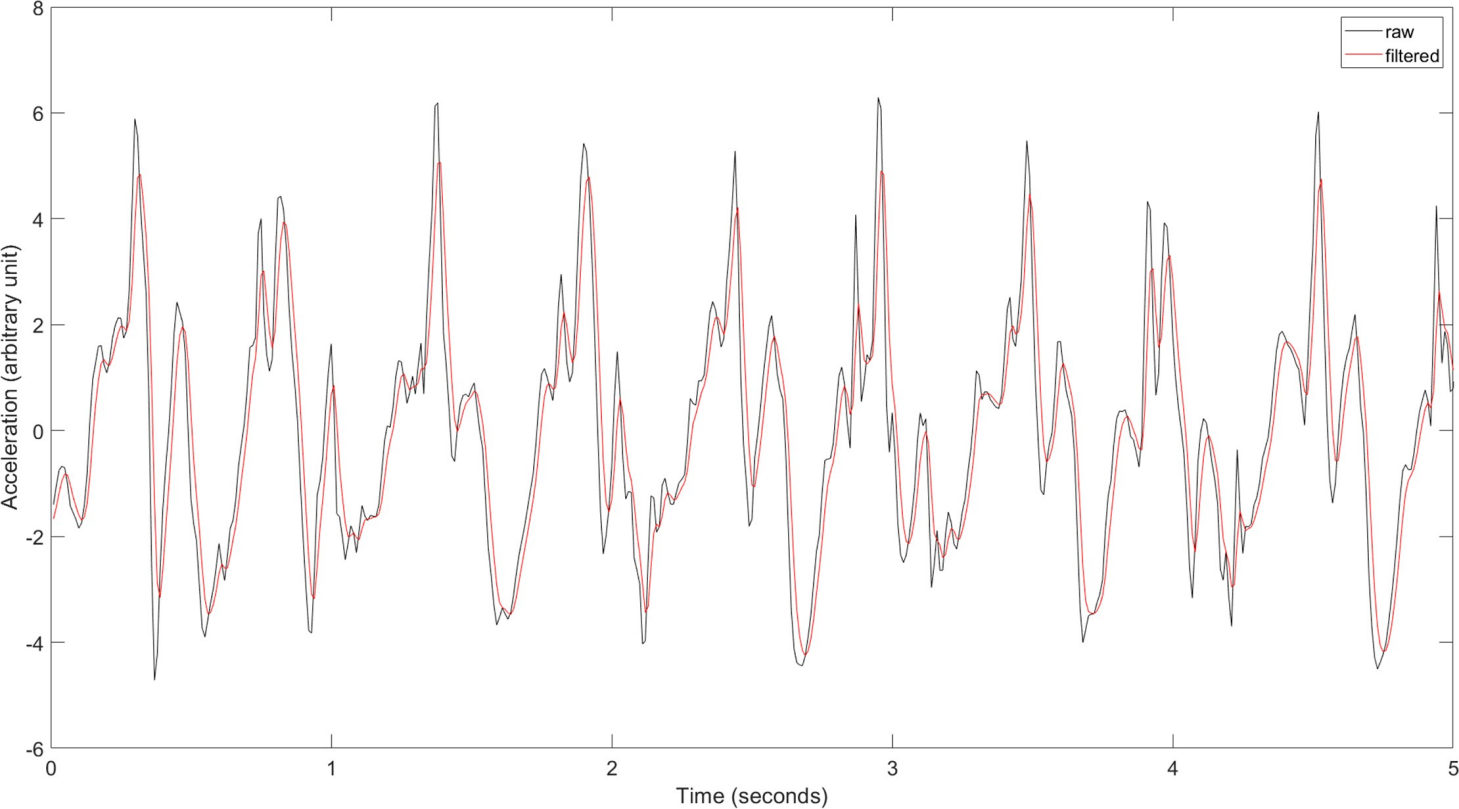
Example signal from raw ang low-pass filtered walking signal, from the x-axis accelerometer.

In the beginning of the data processing, only recordings that included both acceleration and gyroscope data files were selected for preprocessing. Out of 97 participants, 80 subjects had the complete dataset for at least one recording of analyzable walking data, and in total 146 recordings were successful. The final dataset (n = 58), was selected from these successful recordings by balancing the study and control groups, that is discarding 22 patients with scores 1 or 1.5, as discussed earlier in the Materials section. Example signals of raw and preprocessed data are illustrated in Fig 4 below.

The accelerometer and gyroscope signals collected from each walking test were synchronized by removing any excessive parts of the beginning of the files, such that the walking tests were aligned from each sensor. This operation also removed the part of the signals not belonging to the actual test. Both signals included three channels for x-, y- and z-axis (Fig 3). The signals were then interpolated at a sampling frequency of 100 Hz, since the phone sensors were using slightly asynchronous sampling. Then, all signals were smoothed with a fourth order low-pass Butterworth filter with a cutoff frequency at 20 Hz, earlier used in gait analysis studies by Zhu et al. [35]. Also, it has been shown that meaningful human movement is below 20 Hz [36], thus justifying the selected cut-off frequency.

### Segmentation

The relevant information of gait is within individual steps and in the combination of several consecutive steps. This study aimed to differentiate the walking features of PD patients and control subjects within single steps, and then to classify the subjects into these two classes based on the majority of the classified steps. Thus, a stride detection algorithm was developed based on and modifying the earlier work by Haladjian et al. [31] to identify each step of the walking segment. The aim of the walking detection was to detect individual steps and to reject the inactive periods from the beginning and the end of the measurements.

**Fig 5:**
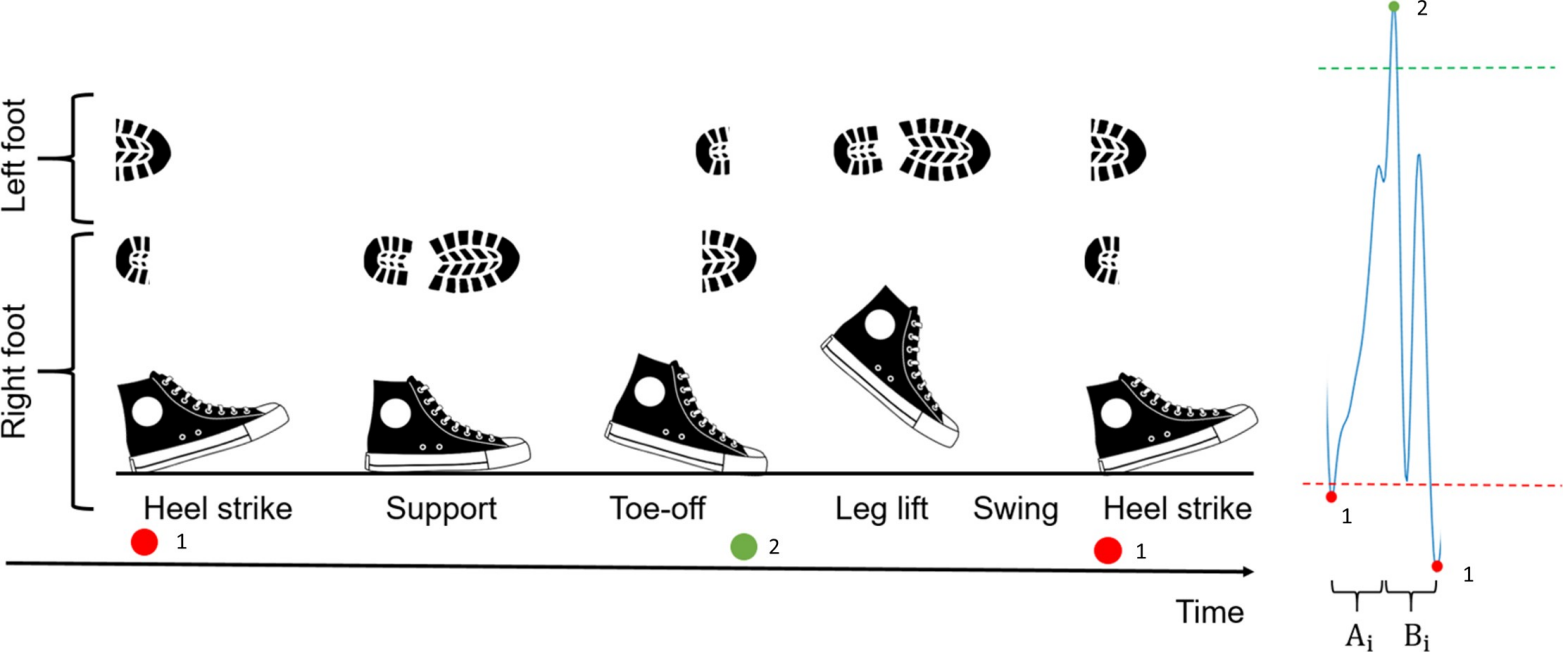
One step cycle from a filtered accelerometer signal in the vertical direction (from heel strike to heel strike). The peaks (green dot) and troughs (red dots) were identified as the local maxima and minima above and below a threshold, respectively. Thresholds are indicated by the green and red dotted lines. Two segments, A_i_ and B_i_ were measured for each step *i*. A denotes the time from the beginning of a heel strike to a toe-off. B denotes the time from the toe-off to the next heel strike.

The vertical direction (x-axis) of the acceleration signal recorded in each walking test was used to detect the beginning of each step. A heel strike, illustrated in Fig 5 with a red dot, translates to the signal as a sharp, negative acceleration in the signal. The local maxima in the signal were also considered. These were corresponding to the toe-off stage of a step, where one foot was just about to leave the ground. Detecting the local maxima and minima of the signal produced a more accurate measure of the duration of ground phase of each step.

To distinguish the walking segments from the inactive period, a wavelet transformation was applied to the x-axis (vertical) of the acceleration signal. This made it easier to visualize the minima and maxima of the signal waveform. Peaks were detected as local maxima that were above a positive acceleration threshold, and troughs were detected as local minima that were below a negative acceleration threshold. Generation of a typical pattern of accelerometer signal in vertical direction during one step is illustrated in Fig 5.

After all peaks and troughs were identified, two segments were measured per step cycle. For each step *i*, the time from minimum to maximum stride amplitude (A_i_), and the time from maximum to minimum stride amplitude (B_i_) were measured. The mean duration of these segments was calculated from all the steps, and the length of the step was a fixed window from the sum of A and B. This approach was based on an earlier study [31]. This process was performed to all walking recordings. This implementation for walking is developed with a certainty, that the recorded files only contain walking and/or standing, and therefore no other types of activity are present to disturb the stride detection.

### Feature extraction and selection

Once individual steps were segmented from walking tests, 201 statistical features were calculated for each step. These features included for example, mean, median, and standard deviation calculated for every axis of each signal as well as comparative features. Features comparing the recorded signals, such as correlation between two axes, were calculated between ACC and GYRO signals on different axes (for example, ACC-X and GYRO-Y), or different axes of the same sensor (for example, ACC-X and ACC-Z). Similar features have been used in previous PD detection studies as well, for example by Mehrang et al. [28] and Arora et al. [6]. All the calculated features are listed in the S1 Table.

Feature selection is often used to pick the best features from a large initial set of features thus decreasing the computational demand and improving generalization of the machine learning method. Three feature selection methods were compared in the study: minimum Redundancy Maximum Relevance (mRMR) [37], sequential forward feature selection (SFS), and sequential backward feature selection (SBS) [38].

In general, maximum relevance methods select features based on maximum mutual information by adding more features in the subset. However, they may often contain material that is redundant in the selected subset. Minimum Redundancy Maximum Relevance (mRMR) optimizes the relevance and minimizes the redundancy of selected features. MRMR compares mutual information of two features and aims to choose the ones that affect the result the most (maximum relevance) but they are still different enough (minimum redundancy) [37]. The mRMR algorithm has been used in studies related to classifying pedestrian information [39], identifying physical activities [40] and recognizing early PD from voice recordings [41] and therefore, it could be suitable for assessing gait features of PD patients as well.

The mRMR selection calculates the best features based on the desired number of features given as an input, and the optimal features are selected during the execution of the algorithm. Hence, mRMR must be run several times, each time specifying a desired number of features, to determine the optimal set of features. This can make mRMR less efficient, as finding the optimal number of features may involve running mRMR on every possible feature set size in the feature space. The optimal feature set was found by looking 20 separate trials of mRMR, with the number of features derived ranging from five to 100 in increments of five. These feature sets were tested by using the Support Vector Machine (SVM), logistic regression, and linear discriminant analysis (LDA). SVM has been referenced in earlier classification studies related to PD [24, 42], and the other two classifiers were selected to study, whether the type of classifier has a noticeable effect in the feature number selection.

Sequential feature selection is based on sequentially adding or discarding features, until the preset criterion does not change, and the classification result is “optimal”. Sequential feature selection may run either forward or backward direction. Forward selection (SFS) starts with one feature and sequentially adds new features by evaluating, which feature improves the result the most, whereas SBS starts with all features and discards them one by one as long as there is an improvement in prediction. Feature selection was performed individually for each classifier thus obtaining the best sets of features for that particular classifier based on the two methods.

### Classification and validation

We applied nine different machine learning algorithms to classify the steps to PD or control steps based on the features selected in the previous step. The classification algorithms were:

- Classification tree
- Gaussian Kernel
- Linear discriminant analysis (LDA)
- Ensemble
- k Nearest neighbours (kNN, k = 49)
- Logistic regression
- Naive Bayes
- Support Vector Machine (SVM)
- Random Forest (RF)

We selected these methods, since they can be considered as basic supervised learning algorithms. Most of them have been used in the existing literature: SVM [24, 42], neural network [5], Naive Bayes [43], logistic regression [44], kNN [45], LDA [22, 23], and RF [26, 27]. These methods were applied the feature sets selected using SFS, SBS, and mRMR.

Two classifiers, namely kNN and RF required some additional setting and methods before they could be applied. In the kNN method, the square root of the training set size has been proposed as the rule for selecting the value of k [46], and an odd number should be used in binary classification to avoid a tied vote. Based on these principles, we selected k = 49 for this study. Some studies have also suggested that k should be between 1–10 [45], but in our study the selection of k = 49 provided better results. In general, the higher is the k, the more generalizable is the model and less overfitting it contains. RF has been implemented in two ways: first by utilizing all 201 features in the classification, and then by recalculating the classification with 20 most important features selected by the RF classifier. In addition, mRMR features were used for RF as well. As the RF inherently ranks the features in order, it is not sensible to use the SFS or SBS feature selection methods with it.

The performance of the classifiers was evaluated using a leave-one-out cross validation for each subject, where in each iteration the steps of all but one subject were used to train the classifier, and the remaining subject’s steps were used to test the classifier [47]. Eight of the classifiers were used together with the three feature selection methods, and thus the feature selection approaches were compared simultaneously. RF was used by repeating the leave-one-subject out method 11 times for each subject, and the results were calculated as an average of the classifications. 11 repetitions ensured, that there can be no tied votes when classifying the steps and that the effect of variability of the RF algorithm is minimized.

The performance of the methods was then tested by comparing the overall accuracy, as well as the sensitivity and specificity of the classification. Accuracy is the ratio of correctly classified steps to all steps. In this study, sensitivity means the accuracy of detecting true PD steps (the ratio of correctly classified PD steps), and specificity means the accuracy of detecting true control steps (the ratio of correctly classified control steps in the testing data).

Additionally, the performance was evaluated subject by subject, by further classifying the subject into PD or control group based on majority of the classified steps. Thus, if more than 50% of the steps were classified as PD steps, the whole subject is classified as a PD patient, and vice versa. If misclassifications occurred, the collected health information of the subjects was checked to identify and understand possible explanations for misclassification. The results of this step are presented for the highest performing classifiers selected by the individual step classification.

Statistical testing was performed to the classifiers to study the significance of the differences in the results. Firstly, a non-parametric Cochran’s Q test was used to test the significance of a) individual step classifications with nine different classifiers, and b) the overall classification of the subjects (majority of the steps classified in the correct category). The null hypothesis is that there is no statistical difference between the classifiers in the classification accuracy. The null hypothesis is rejected, if the p < 0.01. A post-hoc comparison of classifier pairs was performed with an additional Dunn’s test.

## Results

### Feature selection

**Fig 6:**
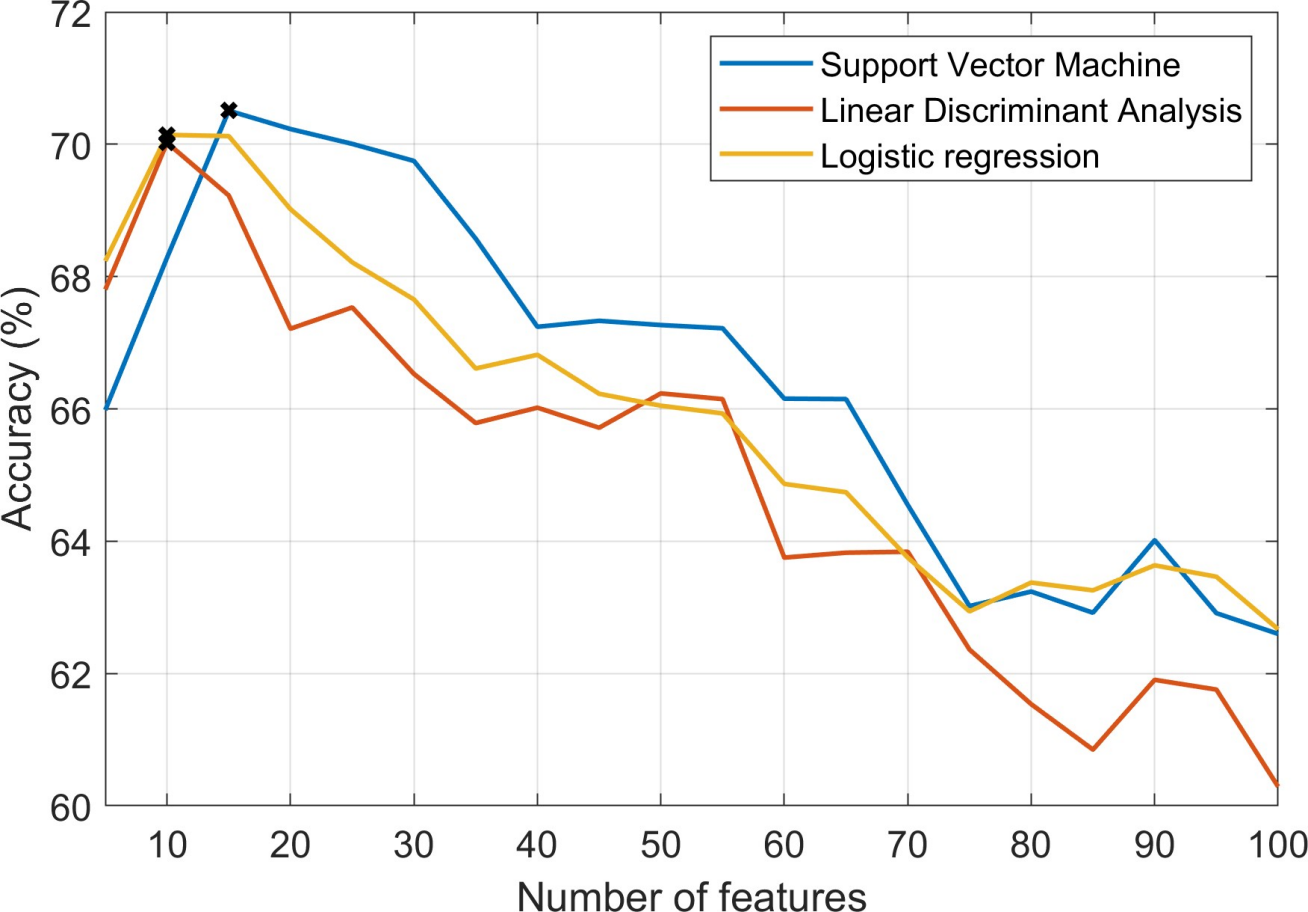
Accuracy plotted for 5–100 features selected with the mRMR algorithm and using the Support Vector Machine classifier, logistic regression and linear discriminant analysis. The highest result for each classifier is marked with an x.

The optimal number and set of features found by the mRMR algorithm was tested as discussed in the Methods section. The overall results between 5 to 100 features with SVM, logistic regression, and LDA are presented in Fig 6.

This comparison resulted in the best results between 5 and 20 features. The detailed values for the classifiers and feature selection methods are illustrated in Table 2. With all three classifiers, the accuracy started to decrease when more than 20 features were selected However, the optimal number of features was not the same for all the classifiers or the performance metrics. The changes were, however, rather small which can be seen in Table 2.

**Table 2:** Detailed classification results (accuracy) obtained with Support Vector Machine, logistic regression, and linear discriminant analysis for minimum Redundancy Maximum Relevance sets between 5–20 features.

Number of features	Support Vector Machine	Linear Discriminant Analysis	Logistic regression
**5**	66.0%	67.8%	68.2%
**10**	68.3%	70.0%	70.1%
**15**	70.5%	69.2%	70.1%
**20**	70.2%	67.2%	69.0%

The highest overall accuracy was found for 15 features using SVM, 10 features using LDA, and 10 or 15 features using logistic regression. Therefore, 15 was chosen as the final number of selected features for mRMR for all the classifiers, and it was used further in this study. The overall number of selected features in each method is summarized in Table 3.

**Table 3:** Number of selected features with different classification and feature selection methods.

	Number of features		
Classifier	mRMR	Sequential FFS	Sequential BFS
**Linear discriminant analysis**	15	8	169
**Ensemble**	15	6	187
**Gaussian Kernel**	15	5	199
**K Nearest Neighbours**	15	4	174
**Logistic regression**	15	7	200
**Naive Bayes**	15	7	143
**Support Vector Machine**	15	5	
**Classification tree /Decision tree**	15	5	86
**Random Forest (all features)**	201		
**Random Forest (20 best features)**	20		
**Random Forest (mRMR features)**	15		

Table 3 shows that in SFS the number of selected features is 4–8, whereas SBS selection has selected more features, between 86–200. SBS calculation was computationally challenging. For example, with SVM classifier the analysis took several weeks even with a high-performance PC. This can be compared to mRMR, which lasted a few minutes, RF took only 8 hours when using all the features, and SFS for all classifiers around 24 hours. Therefore, the computational needs of these algorithms are very different.

In RF classification, the algorithm was first implemented with all 201 features. After the first round, 20 most significant features decided by the algorithm were selected, and the classification was performed again. The algorithm was also tested with features selected with mRMR method.

### Classification methods

The results for nine different classifiers with three different feature selection methods are shown in Table 4.

**Table 4:** Classification of individual steps with nine classifiers and three feature selection methods.

	Accuracy			Sensitivity			Specificity		
Classifier	mRMR	SFS	SBS	mRMR	SFS	SBS	mRMR	SFS	SBS**
**Linear discriminant analysis**	69.2%	75.0%	65.8%	74.8%	80.1%	66.0%	63.7%	69.8%	65.5%
**Ensemble**	68.2%	70.6%	64.1%	65.6%	68.1%	60.4%	70.8%	73.0%	67.8%
**Gaussian Kernel**	47.8%	71.7%	43.5%	49.0%	74.5%	46.2%	46.7%	68.9%	40.7%
**K Nearest Neighbours**	66.6%	75.1%	62.1%	65.6%	75.9%	58.2%	67.6%	74.2%	66.1%
**Logistic regression**	70.1%	74.6%	67.9%	72.9%	79.0%	72.3%	67.3%	70.2%	63.6%
**Naive Bayes**	71.3%	75.3%	66.3%	87.8%	83.7%	87.8%	54.8%	66.9%	44.8%
**Support Vector Machine**	70.5%	74.3%	69.3%	75.7%	83.2%	66.3%	65.3%	65.4%	72.2%
**Classification tree /Decision tree**	66.0%	66.7%	71.5%	63.4%	66.1%	64.1%	68.6%	67.3%	79.0%
Random Forest*:									
Random Forest (all features)	67.9%			62.8%			72.9%		
Random Forest (20 best features)	71.4%			68.2%			74.6%		
Random Forest (mRMR features)	71.7%			70.4%			72.9%		

* Random forest was calculated with all features, 20 best features and mRMR features. SFS and SBS were not used for it.

**Abbreviations: mRMR = minimum Reduncancy, Maximum Relevance.

SFS = Sequenctial Forward feature Selection.

SBS = Sequential Backward feature Selection.

The highest accuracy was obtained using Naive Bayes classifier with SFS (75.3%), highest sensitivity also with Naive Bayes using mRMR (87.8%), and highest specificity with kNN and SFS (74.2%). Although Naive Bayes + SFS combination had the highest accuracy and sensitivity (of the SFS features), it had lower specificity than many other classifiers. The worst performing classifier seems to be the Gaussian Kernel with SBS which had all three performance metrics less than 50%.

**Fig 7:**
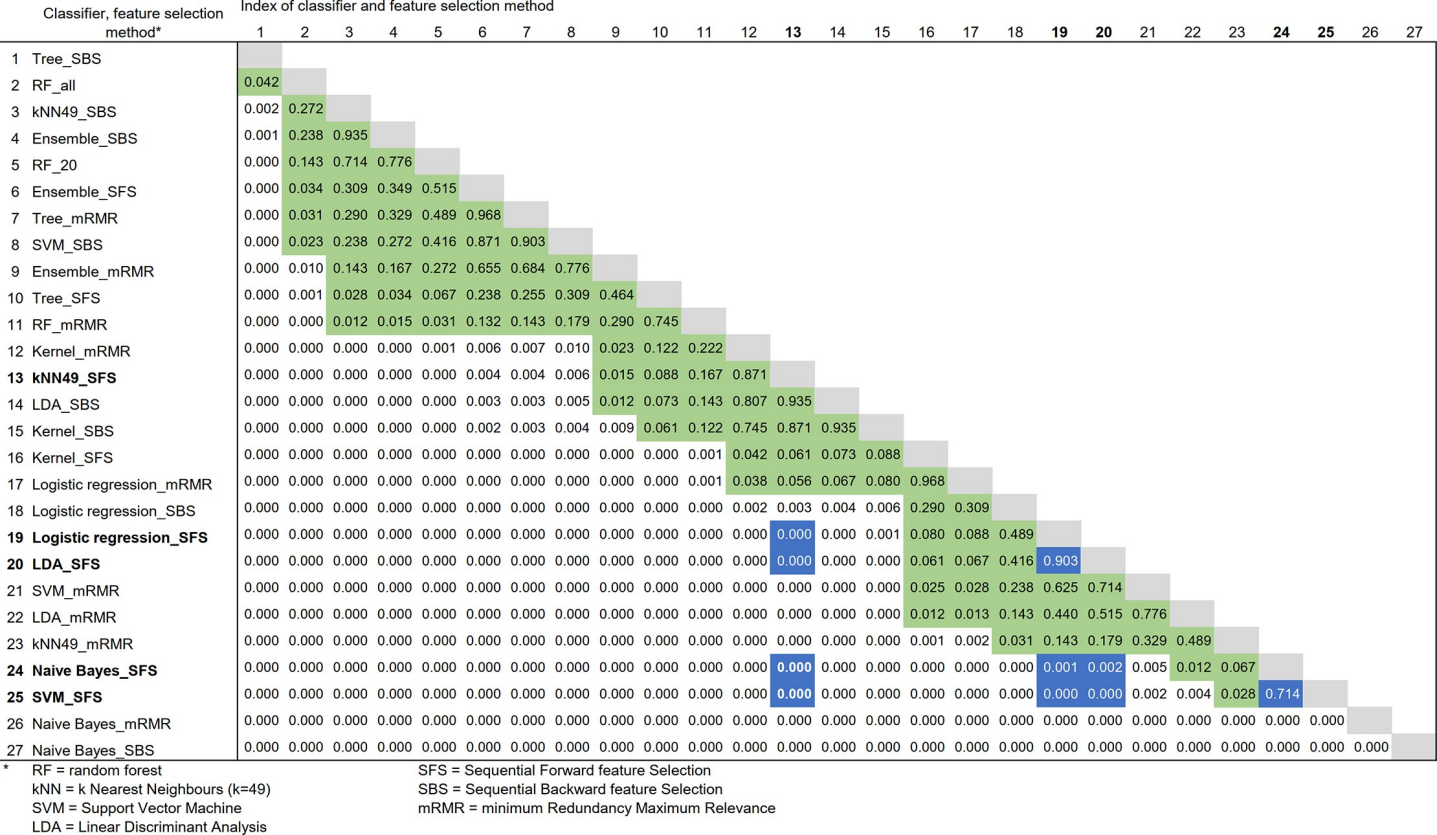
Statistical testing results for Cochran’s Q test and post-hoc test with pairwise comparison of individual step classification. P-values above 0.01 are marked with green background, and the best five classifiers compared are marked with blue background and white text. The classifier names with the highest accuracy are also bolded. The classifier pairs are placed in the order of the p-value.

The statistical significance of the comparison of classifiers was tested with Cochran’s Q test. As Cochran’s Q test showed statistical significance (p<0.01), Dunn’s post-hoc test was applied for pairwise comparison of the classifiers. The results of the post-hoc statistical test are in Fig 7.

Fig 7 shows, that most of the pairwise comparisons have a p-value of p < 0.01, meaning that the results of the classifier pairs are not statistically similar. Five classifiers with highest accuracy have been highlighted with blue background, and from them we can notice that logistic regression and LDA have high similarity (p-value = 0.903), as well as Naive Bayes and SVM (p-value = 0.714). However, kNN does not appear to be similar to any of the other high performing classifiers.

In the next phase, the overall classification of subjects was performed by major voting principle. Classifiers for this phase were selected from the classifiers having the highest accuracy. Classifications were performed with feature sets selected with SFS method. The results of the overall classification of the five best performing classifiers are shown in Table 5.

**Table 5:** Overall classification of subjects based on classification of individual steps and majority voting. Five highest performing classifiers were selected for comparison of overall classification. Columns on the right side present the number of subjects classified in each category.

Classifier	Accuracy	Sensitivity	Specificity	True positive	True negative	False positive	False negative	Sum
**K-Nearest Neighbours (k = 49)**	84.5%	88.5%	81.3%	23	26	6	3	58
**Linear discriminant analysis**	81.0%	78.1%	84.6%	25	22	4	7	58
**Logistic regression**	81.0%	78.1%	84.6%	25	22	4	7	58
**Naive Bayes**	77.6%	75.0%	80.8%	24	21	5	8	58
**Support Vector Machine**	74.1%	70.6%	79.2%	24	19	5	10	58

The highest accuracy of subject-wise classification was obtained with kNN (84.5% accuracy). LDA and logistic regression performed equally well (81% accuracy). The misclassification rate for the kNN was 15.5%, and for the other two 19.0%. The last two classifiers presented in Table 5 had lower accuracy and higher misclassification rate. Cochran’s Q test showed, that there was no statistical difference between these classifiers in the overall classifications (p-value = 0.203).

## Discussion

The best performing classifier in the overall classification was kNN achieving accuracy, sensitivity and specificity of 84.5%, 88.5%, and 81.3%, respectively. The other classifiers from the best five all obtained better results than what was achieved in an earlier study by Mehrang et al. [28]. Although our walking tests were based on the same mPower protocol, in our case, the physiotherapist controlled the sensors and calculated the steps instead of the subject themselves as in [28]. Also, excluding the mild stages of the disease (UPDRS score 1 and some of 1.5) might have an effect on the results.

Compared to the results received by Arora et al. [6], our larger dataset might have brought more variation to the PD symptoms. They received an average accuracy of 98.5% and a specificity of 97.5%, by conducting multiple walking tests and posture tests daily and performed by 20 subjects, whereas we used only controlled walking tests. However, a collection of 20 subjects is a rather small representation of the PD symptoms to be used in machine learning applications. It should also be noted, that while in Arora’s study [6] RF classifier provided significantly better results than the other classifiers, this was not the case in our analysis.

The number of features obtained with different feature selection methods varied a lot. The number of features selected by SFS method varied between 4–8, whereas with the features selected by mRMR method, the best results were obtained with fifteen features. On the other hand, SBS selected as many as 200 features. However, the best accuracy and sensitivity were obtained with the 7 features selected by SFS and Naive Bayes classifier, and 4 features and kNN classifier when considering the overall classification. Hence, using a small subset of features kept the algorithm computationally efficient and provided the best classification results. Using an excessive number of features would both slow the calculation and decrease the performance due to model overfitting. We also noticed, that running the SBS algorithm was computationally very demanding, and it did not provide more accurate results than mRMR or SFS. Therefore, in this dataset, the use of SBS in further studies is not recommended.

We did not interfere with the medication schedules or dosing for PD patients in this study. Therefore, some misclassifications of PD patients may have occurred, if they took their medication just before the study. The amount of false negative overall classification was the lowest with kNN classifier using SFS, in which three patients were classified as non-PD.

Three PD-patients and three control subjects were classified incorrectly with all three classifiers with the highest overall accuracy (kNN, LDA, and logistic regression). Two of the control subjects had gait affecting diagnoses, including arthritis, fibromyalgia, osteoporosis and an old hip fracture. The third one had some diagnoses including orthostatic hypotension, but no clear reason for misclassification. Regarding the misclassified PD patients, two out of three were diagnosed only one year before the study, and the third one had taken the levodopa medication only one hour before the study. Therefore, their misclassification could be due to these findings.

The limitations of this study should be noted when interpreting these results. Since the mean age of PD patients and controls was almost 70 and 60 years, respectively, both groups had several additional health issues that might have had an effect in the walking tests. Therefore, it is a challenge to identify which statistical features are describing PD, and which are related to issues in movement in general. Higher age and additional diagnoses may also increase the misclassification rate. However, misclassifications also happen in the traditional process of diagnosing PD and separating the disease from similar conditions [7].

Another limitation or a challenge is to transfer the methods based on clinic data to recordings measured in a wild environment. The future aim of applying these methods is in the analysis of walking segments performed in free-living conditions at home. However, in order to analyze relevant data, the walking segments need to be reliably detected. The current walking detection algorithm suits well for clinic data, since we know that the 20 step walking tests only contain walking or standing and thus, it is easy to extract the steps from the signal. However, when we analyze the data collected from free-living environment, the signal may contain any movement performed by the subject with or without an assistive device, such as a car, bicycle or an elevator. Therefore, the walking recognition needs to be carefully designed and its reliability verified.

Based on the results and literature discussed earlier in this work, the smartphone proves to be a feasible device for measuring the symptoms of PD. It provides relevant accuracy for collecting data with an inexpensive system, but it also allows the subject to move freely. Therefore, it would be more convenient in remote studies compared to video systems or expensive wearables. The results could be generalized to other datasets as well recorded with a smartphone attached to the waist.

Further work with this dataset will include studying the classification of the PD patients into several categories based on their UPDRS score. This classification could be used to detect whether the patients are under the levodopa medication or not. When more control subjects have been collected, we can also include the PD patients with scores 1 and 1.5 into the analysis. The long-term aim is to build an automated analysis system for assessing the daily variations of PD at home without disturbing the patient in their everyday life.

In the future, automated measurement systems could be used to monitor patients with motor difficulties and provide an objective view to fast changing symptoms in free-living settings. These traditional, computationally inexpensive classification approaches could for example, enable the whole signal processing and data analysis chain being implemented into a mobile device application thus providing an efficient tool for improving the quality of treatment e.g. by better adjustment of medication. As healthcare service resources are limited, the use of remote and mobile applications to store relevant information for planning effective care is important.

## Conclusion

This study demonstrates the potential of using inertial sensors integrated in a smartphone combined with machine learning classification methods to identify patients with PD. We compared several methods in feature selection and classification phases of the machine learning pipeline and obtained results that are with results found in prior literature.

The research question of this study was to determine, what are feasible ways of selecting the features and classifying the walking tests performed at the clinic. The most feasible feature selection method for this dataset was SFS that selected 4–8 features depending on the classifier and resulted in the highest accuracy in classifying both individual steps and overall subjects. Individual step classification was most accurate (75.3%) with Naive Bayes, but the overall classification was the most accurate (84.5%) with the kNN algorithm (with k = 49).

These accuracies are comparable to earlier studies found in the literature. The earlier literature has shown slightly higher accuracies for measurements performed at clinics or research laboratories [22], and smaller datasets [6], but a lower accuracy for completely independent home measurements [28]. Even though RF has been found very efficient in earlier studies [6], it did not perform particularly well in our analysis. Evaluation of the methods found useful in this study in further analyses of this dataset is therefore justified. Future work includes the application of these methods into a free-living dataset, and the recognition of symptom changes from walking. Also, relevant walking detection algorithms for free living data should be investigated.

## Supporting information

S1 Table Features calculated from the dataset. All features, including statistical and comparative features and their short explanations.
(DOCX)

S2 Table Feature selection results. Selected features with the four selection methods: minimum Redundancy Maximum Relevance (mRMR), and sequential forward and backward feature selection (SFS and SBS, respectively), and 20 most important features selected in Random Forest (RF).
(XLSX)
