# Supplementary material for: Parkinson’s disease detection from 20-step walking tests using inertial sensors of a smartphone: Machine learning approach based on an observational case-control study
Source: PLoS One. 2020 Jul 23;15(7):e0236258. doi: 10.1371/journal.pone.0236258 (PMC7377496; doi:10.1371/journal.pone.0236258)
Supplement: S1 Table — All features, including statistical and comparative features and their short explanations. (DOCX) [file pone.0236258.s001.docx]

| **Feature abbreviation** | **Feature name** | **Variable** | **Channel** |
| --- | --- | --- | --- |
| minAx | minimum | acceleration | X |
| minAy | minimum | acceleration | Y |
| minAz | minimum | acceleration | Z |
| minGx | minimum | gyroscope | X |
| minGy | minimum | gyroscope | Y |
| minGz | minimum | gyroscope | Z |
| minMA | minimum | acceleration | Magnitude |
| minMG | minimum | gyroscope | Magnitude |
| maxAx | maximum | acceleration | X |
| maxAy | maximum | acceleration | Y |
| maxAz | maximum | acceleration | Z |
| maxGx | maximum | gyroscope | X |
| maxGy | maximum | gyroscope | Y |
| maxGz | maximum | gyroscope | Z |
| maxMA | maximum | acceleration | Magnitude |
| maxMG | maximum | gyroscope | Magnitude |
| meanAx | mean | acceleration | X |
| meanAy | mean | acceleration | Y |
| meanAz | mean | acceleration | Z |
| meanGx | mean | gyroscope | X |
| meanGy | mean | gyroscope | Y |
| meanGz | mean | gyroscope | Z |
| meanMA | mean | acceleration | Magnitude |
| meanMG | mean | gyroscope | Magnitude |
| varAx | variance | acceleration | X |
| varAy | variance | acceleration | Y |
| varAz | variance | acceleration | Z |
| varGx | variance | gyroscope | X |
| varGy | variance | gyroscope | Y |
| varGz | variance | gyroscope | Z |
| varMA | variance | acceleration | Magnitude |
| varMG | variance | gyroscope | Magnitude |
| stdAx | standard deviation | acceleration | X |
| stdAy | standard deviation | acceleration | Y |
| stdAz | standard deviation | acceleration | Z |
| stdGx | standard deviation | gyroscope | X |
| stdGy | standard deviation | gyroscope | Y |
| stdGz | standard deviation | gyroscope | Z |
| stdMA | standard deviation | acceleration | Magnitude |
| stdMG | standard deviation | gyroscope | Magnitude |
| medianAx | median | acceleration | X |
| medianAy | median | acceleration | Y |
| medianAz | median | acceleration | Z |
| medianGx | median | gyroscope | X |
| medianGy | median | gyroscope | Y |
| medianGz | median | gyroscope | Z |
| medianMA | median | acceleration | Magnitude |
| medianMG | median | gyroscope | Magnitude |
| trapzAx | trapezoid | acceleration | X |
| trapzAy | trapezoid | acceleration | Y |
| trapzAz | trapezoid | acceleration | Z |
| trapzGx | trapezoid | gyroscope | X |
| trapzGy | trapezoid | gyroscope | Y |
| trapzGz | trapezoid | gyroscope | Z |
| trapzMA | trapezoid | acceleration | Magnitude |
| trapzMG | trapezoid | gyroscope | Magnitude |
| aavAx | Average absolute Acceleration variation | acceleration | X |
| aavAy | Average absolute Acceleration variation | acceleration | Y |
| aavAz | Average absolute Acceleration variation | acceleration | Z |
| aavGx | Average absolute Acceleration variation | gyroscope | X |
| aavGy | Average absolute Acceleration variation | gyroscope | Y |
| aavGz | Average absolute Acceleration variation | gyroscope | Z |
| aavMA | Average absolute Acceleration variation | acceleration | Magnitude |
| aavMG | Average absolute Acceleration variation | gyroscope | Magnitude |
| madAx | mean amplitude deviation | acceleration | X |
| madAy | mean amplitude deviation | acceleration | Y |
| madAz | mean amplitude deviation | acceleration | Z |
| madGx | mean amplitude deviation | gyroscope | X |
| madGy | mean amplitude deviation | gyroscope | Y |
| madGz | mean amplitude deviation | gyroscope | Z |
| madMA | mean amplitude deviation | acceleration | Magnitude |
| madMG | mean amplitude deviation | gyroscope | Magnitude |
| iqrAx | inter-quantile range | acceleration | X |
| iqrAy | inter-quantile range | acceleration | Y |
| iqrAz | inter-quantile range | acceleration | Z |
| iqrGx | inter-quantile range | gyroscope | X |
| iqrGy | inter-quantile range | gyroscope | Y |
| iqrGz | inter-quantile range | gyroscope | Z |
| iqrMA | inter-quantile range | acceleration | Magnitude |
| iqrMG | inter-quantile range | gyroscope | Magnitude |
| skewnessAx | skewness | acceleration | X |
| skewnessAy | skewness | acceleration | Y |
| skewnessAz | skewness | acceleration | Z |
| skewnessGx | skewness | gyroscope | X |
| skewnessGy | skewness | gyroscope | Y |
| skewnessGz | skewness | gyroscope | Z |
| skewnessMA | skewness | acceleration | Magnitude |
| skewnessMG | skewness | gyroscope | Magnitude |
| kurtosisAx | kurtosis | acceleration | X |
| kurtosisAy | kurtosis | acceleration | Y |
| kurtosisAz | kurtosis | acceleration | Z |
| kurtosisGx | kurtosis | gyroscope | X |
| kurtosisGy | kurtosis | gyroscope | Y |
| kurtosisGz | kurtosis | gyroscope | Z |
| kurtosisMA | kurtosis | acceleration | Magnitude |
| kurtosisMG | kurtosis | gyroscope | Magnitude |
| rmsAx | root-mean-square | acceleration | X |
| rmsAy | root-mean-square | acceleration | Y |
| rmsAz | root-mean-square | acceleration | Z |
| rmsGx | root-mean-square | gyroscope | X |
| rmsGy | root-mean-square | gyroscope | Y |
| rmsGz | root-mean-square | gyroscope | Z |
| rmsMA | root-mean-square | acceleration | Magnitude |
| rmsMG | root-mean-square | gyroscope | Magnitude |
| quantileAx1 | 1st quantile | acceleration | X |
| quantileAx2 | 2nd quantile | acceleration | X |
| quantileAx3 | 3rd quantile | acceleration | X |
| quantileAx4 | 4th quantile | acceleration | X |
| quantileAy1 | 1st quantile | acceleration | Y |
| quantileAy2 | 2nd quantile | acceleration | Y |
| quantileAy3 | 3rd quantile | acceleration | Y |
| quantileAy4 | 4th quantile | acceleration | Y |
| quantileAz1 | 1st quantile | acceleration | Z |
| quantileAz2 | 2nd quantile | acceleration | Z |
| quantileAz3 | 3rd quantile | acceleration | Z |
| quantileAz4 | 4th quantile | acceleration | Z |
| quantileGx1 | 1st quantile | gyroscope | X |
| quantileGx2 | 2nd quantile | gyroscope | X |
| quantileGx3 | 3rd quantile | gyroscope | X |
| quantileGx4 | 4th quantile | gyroscope | X |
| quantileGy1 | 1st quantile | gyroscope | Y |
| quantileGy2 | 2nd quantile | gyroscope | Y |
| quantileGy3 | 3rd quantile | gyroscope | Y |
| quantileGy4 | 4th quantile | gyroscope | Y |
| quantileGz1 | 1st quantile | gyroscope | Z |
| quantileGz2 | 2nd quantile | gyroscope | Z |
| quantileGz3 | 3rd quantile | gyroscope | Z |
| quantileGz4 | 4th quantile | gyroscope | Z |
| quantileMA1 | 1st quantile | acceleration | magnitude |
| quantileMA2 | 2nd quantile | acceleration | magnitude |
| quantileMA3 | 3rd quantile | acceleration | magnitude |
| quantileMA4 | 4th quantile | acceleration | magnitude |
| quantileMG1 | 1st quantile | gyroscope | magnitude |
| quantileMG2 | 2nd quantile | gyroscope | magnitude |
| quantileMG3 | 3rd quantile | gyroscope | magnitude |
| quantileMG4 | 4th quantile | gyroscope | magnitude |
| zrcAx | Zero-crossing rate | Acceleration | X |
| zrcAy | Zero-crossing rate | Acceleration | Y |
| zrcAz | Zero-crossing rate | Acceleration | Z |
| accelMagnitudeOctant | magnitude octant | Acceleration | Magnitude |
| rotationMagnitudeOctant | magnitude octant | Gyroscope | Magnitude |
| smaAcceleration | Simple moving average | Acceleration |  |
| smaRotation | Simple moving average | Gyroscope |  |
| svmAcceleration | Signal vector magnitude | Acceleration |  |
| svmRotation | Signal vector magnitude | Gyroscope |  |
| corrAxAy | correlation | Acceleration | X, Y |
| corrAxAz | correlation | Acceleration | X, Z |
| corrAxGx | correlation | Acceleration, gyroscope | X, X |
| corrAxGy | correlation | Acceleration, gyroscope | X, Y |
| corrAxGz | correlation | Acceleration, gyroscope | X, Z |
| corrAxMA | correlation | Acceleration | X, Magnitude |
| corrAxMG | correlation | Acceleration, gyroscope | X, Magnitude |
| corrAyAz | correlation | Acceleration | Y, Z |
| corrAyGx | correlation | Acceleration, gyroscope | Y, X |
| corrAyGy | correlation | Acceleration, gyroscope | Y, Y |
| corrAyGz | correlation | Acceleration, gyroscope | Y, Z |
| corrAyMA | correlation | Acceleration | Y, magnitude |
| corrAyMG | correlation | Acceleration, gyroscope | Y, magnitude |
| corrAzGx | correlation | Acceleration, gyroscope | Z, X |
| corrAzGy | correlation | Acceleration, gyroscope | Z, Y |
| corrAzGz | correlation | Acceleration, gyroscope | Z, Z |
| corrAzMA | correlation | acceleration | Z, magnitude |
| corrAzMG | correlation | Acceleration, gyroscope | Z, magnitude |
| corrGxGy | correlation | gyroscope | X, Y |
| corrGxGz | correlation | gyroscope | X, Z |
| corrGxMA | correlation | Gyroscope, acceleration | X, magnitude |
| corrGxMG | correlation | Gyroscope | X, magnitude |
| corrGyGz | correlation | Gyroscope | Y, Z |
| corrGyMA | correlation | Gyroscope, acceleration | Y, magnitude |
| corrGyMG | correlation | Gyroscope | Y, magnitude |
| corrGzMA | correlation | Gyroscope, acceleration | Z, Magnitude |
| corrGzMG | correlation | Gyroscope | Z, Magnitude |
| corrMAMG | correlation | Acceleration, gyroscope | Magnitude, magnitude |
| xcorrAxAy | cross correlation | Acceleration | X, Y |
| xcorrAxAz | cross correlation | Acceleration | X, Z |
| xcorrAxGx | cross correlation | Acceleration, gyroscope | X, X |
| xcorrAxGy | cross correlation | Acceleration, gyroscope | X, Y |
| xcorrAxGz | cross correlation | Acceleration, gyroscope | X, Z |
| xcorrAxMA | cross correlation | Acceleration | X, Magnitude |
| xcorrAxMG | cross correlation | Acceleration, gyroscope | X, Magnitude |
| xcorrAyAz | cross correlation | Acceleration | Y, Z |
| xcorrAyGx | cross correlation | Acceleration, gyroscope | Y, X |
| xcorrAyGy | cross correlation | Acceleration, gyroscope | Y, Y |
| xcorrAyGz | cross correlation | Acceleration, gyroscope | Y, Z |
| xcorrAyMA | cross correlation | Acceleration | Y, magnitude |
| xcorrAyMG | cross correlation | Acceleration, gyroscope | Y, magnitude |
| xcorrAzGx | cross correlation | Acceleration, gyroscope | Z, X |
| xcorrAzGy | cross correlation | Acceleration, gyroscope | Z, Y |
| xcorrAzGz | cross correlation | Acceleration, gyroscope | Z, Z |
| xcorrAzMA | cross correlation | acceleration | Z, magnitude |
| xcorrAzMG | cross correlation | Acceleration, gyroscope | Z, magnitude |
| xcorrGxGy | cross correlation | gyroscope | X, Y |
| xcorrGxGz | cross correlation | gyroscope | X, Z |
| xcorrGxMA | cross correlation | Gyroscope, acceleration | X, magnitude |
| xcorrGxMG | cross correlation | Gyroscope | X, magnitude |
| xcorrGyGz | cross correlation | Gyroscope | Y, Z |
| xcorrGyMA | cross correlation | Gyroscope, acceleration | Y, magnitude |
| xcorrGyMG | cross correlation | Gyroscope | Y, magnitude |
| xcorrGzMA | cross correlation | Gyroscope, acceleration | Z, Magnitude |
| xcorrGzMG | cross correlation | Gyroscope | Z, Magnitude |
| xcorrMAMG | cross correlation | Acceleration, gyroscope | Magnitude, magnitude |
